# Efficacy and Safety of Modified Seamless Endoscopic Dacryocystorhinostomy in Patients with Chronic Dacryocystitis

**DOI:** 10.1155/2022/3061859

**Published:** 2022-11-14

**Authors:** Yuchuan Wang, Fang Liu, Miao Cao, Lianfeng Xie, Shuxiang Tan, Linlin Liu

**Affiliations:** ^1^The First Clinical Medical College of Gannan Medical University, Ganzhou, Jiangxi 341000, China; ^2^Department of Ophthalmology, The First Affiliated Hospital of Gannan Medical University, Ganzhou, Jiangxi 341000, China

## Abstract

**Objective:**

To evaluate the efficacy and safety of a modified seamless endoscopic dacryocystorhinostomy (EN-DCR) with chronic dacryocystitis.

**Methods:**

This study included 54 patients (54 eyes) with chronic dacryocystitis treated in our hospital from 2019 to 2021, including 32 patients (32 eyes) who underwent modified and 22 patients (22 eyes) who underwent routine EN-DCR. In the modified EN-DCR, the nasal cavity was filled 30 min before the operation by injection of 1 mg/ml adrenaline hydrochloride and application of ephedrine hydrochloride and nitrofurazone nasal drops. Before the operation, the lacrimal passages were rinsed with a 1 : 2 mixture of dilute methylene blue and normal saline. The “I”-shaped incision was replaced by a ^“^C^“^-shaped incision near the lateral bone window. In place of suturing, a gelatin sponge was applied at the confluence of the lacrimal sac and nasal mucosa. After the end of the operation, the lacrimal sac was filled with tapered expansion sponge for 1 week. In routine EN-DCR, the nasal cavity was filled with 1 mg/ml epinephrine hydrochloride, and nitrofurazone nasal drops were provided for 5 minutes after the beginning of the operation; and a “I”-shaped incision was made in the nasal mucosa, with one stitch for each anterior and posterior flap. Operation time, intraoperative bleeding, and postoperative lacrimal duct irrigation were compared, with the curative effect evaluated after a follow-up of 6 months.

**Results:**

Operation time was significantly shorter (41.3 ± 12.1 min vs. 65.4 ± 11.6 min; *χ*^2^ = 7.312, *P* < 0.05) and intraoperative bleeding was significantly lower (12.5 ± 5.2 ml vs. 60.3 ± 8.9 ml; *χ*^2^ = 24.883, *P* < 0.05) in the modified group than in the routine EN-DCR group. After follow-up for 6 months, the effective cure rate was significantly higher in the modified group than in the routine group (96.9% vs. 68.2%; *χ*^2^ = 6.383, *P* < 0.05).

**Conclusion:**

Compared with routine EN-DCR, modified seamless EN-DCR can achieve better surgical outcomes, shorten operation time, and reduce intraoperative bleeding.

## 1. Introduction

Chronic dacryocystitis is a common disease of the lacrimal passages that is usually caused by stenosis or obstruction of the nasolacrimal ducts. The accumulation of internal secretions in the lacrimal sac leads to overflowing tears and mucous secretion in patients' eyes, with a large number of purulent secretions appearing after secondary infection. Because drug treatment is often ineffective or can only temporarily relieve symptoms, surgical treatment is often required [1]. External dacryocystorhinostomy not only affects a patient's appearance but can also lead to scar hyperplasia, making postoperative effects unsatisfactory. Nasal endoscopy can be used in the diagnosis and treatment of chronic dacryocystitis, with endoscopic dacryocystorhinostomy being a minimally invasive method of reconstructing the lacrimal passages.

The average success rate of endoscopic dacryocystorhinostomy was reported to be 85%. However, there are still some patients who fail the operation. Many factors affect the results of the operation [2], including inappropriate location of the lacrimal sac, inadequate osteotomy, insufficient size of the ostium, inadequate marsupialization, more bleeding during the operation, cicatricial closure of the ostium, potential intranasal pathology, and abnormal anatomical structure. In order to better solve the symptoms of lacrimal overflow, reduce postoperative recurrence, and improve the quality of life of patients, we have further improved the routine of endoscopic dacryocystorhinostomy according to some related factors and achieved good clinical results. The following article describes these improvements and their clinical outcomes.

## 2. Objectives and Methods

This retrospective analysis included 54 eyes of 54 patients with chronic dacryocystitis who were treated in our hospital from June 2019 to December 2021. Thirty-two eyes of 32 patients underwent modified seamless endoscopic dacryocystorhinostomy, whereas 22 eyes of 22 patients underwent routine endoscopic dacryocystorhinostomy. Patients were included if: (1) they had a long-term history of lacrimal discharge with purulent secretion; (2) they showed purulent or mucous reflux on lacrimal duct irrigation; and (3) previous conservative treatment, such as with anti-inflammatory agents, lacrimal duct irrigation, or probing of the lacrimal passage, was ineffective. Patients were excluded if they: (1) had a severe deviation of the nasal septum, as shown by CT dacryocystorhinography; (2) had experienced a traumatic fracture of the nasolacrimal duct, fracture of the orbital bone, rupture of the lacrimal sac, a space-occupying lesion of the nasal cavity, or dacryocystitis; (3) had previously undergone a nasal operation; or (4) were intolerant to operation. This study was approved by the ethics committee of our hospital, and all patients and their families provided written informed consent before operation.

### 2.1. Methods

#### 2.1.1. Routine Endoscopic Dacryocystorhinostomy

Dacryocystography was routinely performed before the operation to roughly determine the location, size, and nasal structure of the lacrimal sac. Operations were performed under general anesthesia. The nasal cavity was filled with cotton that was wetted with 1 mg/ml epinephrine hydrochloride, ephedrine hydrochloride, and nitrofurazone nasal drops 5 minutes after the general anesthesia. Starting from the middle turbinates, the nasal mucosa of the lateral wall of the nasal cavity was cut with a crescent surgical knife. Then the mucosa of the nasal cavity was cut off to form a roughly 8 mm × 10 mm mucoperiosteal flap, exposing the lacrimal bone. An 8 mm × 10 mm bony window was opened with a rongeur to expose the lacrimal sac adequately. Then a crescent-shaped surgical knife was used to make a full-thickness“I”-shaped incision of the lacrimal sac. The anterior and posterior valves of the lacrimal sac were sutured to the corresponding nasal mucosa. The nasal cavity was subsequently filled with cotton balls after coating with tobramycin dexamethasone eye ointment in the lacrimal sac.

#### 2.1.2. Modified Endoscopic Dacryocystorhinostomy

**Figure 1:**
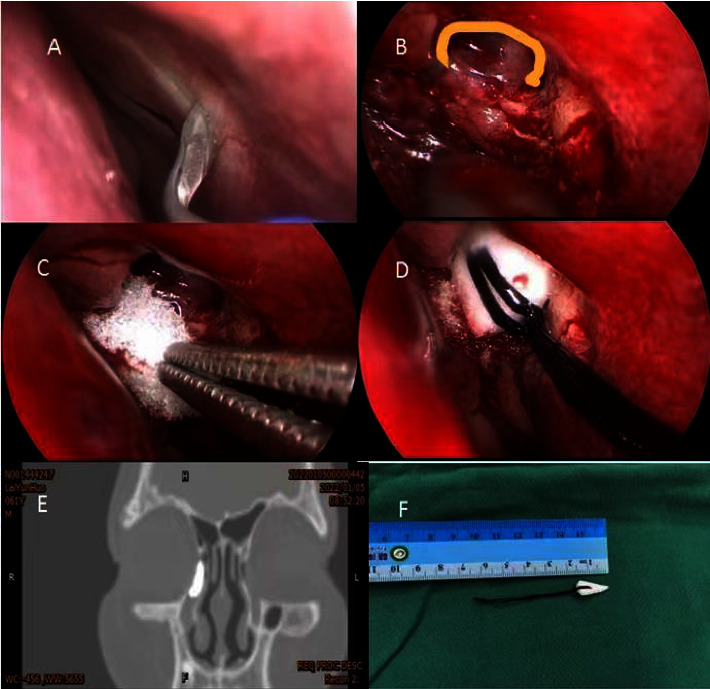
Methods of modified endoscopic dacryocystorhinostomy. (a) Reduction of bleeding from the mucosal incision after filling with 1 mg/ml epinephrine hydrochloride, ephedrine hydrochloride, and nitrofurazone nasal drops. (b) Use of a “C”-shaped lacrimal sac incision rather than an “I”-shaped lacrimal sac incision. (c) Application of a gelatin sponge to the anastomosis of the lacrimal sac and nasal mucosa rather than suturing. (d) Filling of the lacrimal sac with a conical expansion sponge for 1 week. (e) Outcome of dacryocystorhinography. (f) Illustration of a conical expansion sponge.

The procedure used for modified endoscopic dacryocystorhinostomy was similar to that for routine endoscopic dacryocystorhinostomy, except that: (1) the nasal cavity was filled with a cotton which is wet with 1 mg/ml epinephrine hydrochloride, ephedrine hydrochloride, and nitrofurazone nasal drops 30 minutes before the operation; (2) the lacrimal passages were flushed with doubly diluted methylene blue solution before the operation; (3) the traditional “I”-shaped incision was replaced by a “C”-shaped incision near the lateral bone window; (4) applying the gelatin sponge to the confluence of the lacrimal sac and nasal mucosa without the suture; and (5) the lacrimal sac was filled with a conical expansion sponge, which was maintained for 1 week. The expansion sponge was still coated with tobramycin and dexamethasone eye ointment. The results of this procedure are illustrated in Figure 1.

#### 2.1.3. Postoperative Treatment

Postoperatively, patients were instructed to apply 0.5% levofloxacin eye drops four times per day, 0.1% fluorometholone eye drops three times per day, and triamcinolone acetonide nasal spray twice per day. One week after the operation, the expansion sponge was removed from the lacrimal sac, and the lacrimal passage was washed with saline. The lacrimal passage was subsequently washed with a mixture of 5 ml saline and vitamin E (1 tablet) once per week for 1 month, every 2 weeks for the next 2 months, and once per month until 6 months after the operation.

#### 2.1.4. Evaluation Standard of Curative Effect

Cure was defined as the absence of symptoms of lacrimal discharge along with unobstructed irrigation of the lacrimal passage. Improvement was defined as the ability to irrigate the lacrimal passage, partial reflux, partial flow into the nasal cavity, and the absence of secretions. Treatment was defined as ineffective if irrigation of the lacrimal passage was obstructed. The rate of effectiveness was defined as the sum of the cure and improvement rates.

Continuous data were compared by independent sample *t*-tests and categorical data by chi-square tests. All statistical analyses were performed using SPSS20.0 statistical software, with *p* values <0.05 considered statistically significant.

## 3. Results

A comparison of patients who underwent routine and modified endoscopic dacryocystorhinostomy showed no significant differences in age, sex, duration of disease, or lacrimal sac volume (*P* > 0.05 each; Table 1).

A comparison of clinical outcomes in patients who underwent routine and modified endoscopic dacryocystorhinostomy showed that the postoperative effectiveness rate was significantly higher in patients who underwent modified than routine endoscopic dacryocystorhinostomy (96.88% vs. 68.18%; *χ*^2^ = 6.383 *P* < 0.05; Table 2).

Comparisons of operation time and bleeding volume in patients who underwent modified and routine endoscopic dacryocystorhinostomy showed that operation time was significantly shorter (41.3 ± 12.1 min vs. 65.4 ± 11.6 min; *χ*^2^ = 7.312 min, *P* < 0.05) and bleeding volume during the operation significantly lower (12.5 ± 5.2 ml vs. 60.3 ± 8.9 ml; *χ*^2^ = 24.883, *P* < 0.05) in patients who underwent modified endoscopic dacryocystorhinostomy (Table 2).

A comparison of complications in the two groups showed excess bleeding in 11 eyes in the routine endoscopic dacryocystorhinostomy group and in two eyes in the improved endoscopic dacryocystorhinostomy group, with all bleeding effectively controlled by compression and hemostasis. In addition, several patients in both groups experienced headache, fatigue and nasal pain after the operation, with all of these symptoms being relieved after rest. None of the patients in either group experienced serious complications during or after the operation.

## 4. Discussion

Although dacryocystorhinostomy using a nasal endoscope for patients with chronic dacryocystitis was first reported in 1989 and the first clinical study of endoscopic dacryocystorhinostomy was published in 1994, the success rates of early nasal endoscopic operations were not high. Improvements in endoscopic equipment and techniques [3] and further knowledge of lacrimal duct anatomy have increased the usefulness of nasal endoscope technology in ophthalmology [4], with endoscopic dacryocystorhinostomy becoming routine in lacrimal duct operations [5].

Improvements have maximized the success rate of endoscopic dacryocystorhinostomy [6]. These improvements have included nasolacrimal valve suturing; the use of stents [7], mitomycin C, and 5-fluorouracil; and laser-assisted, power terminal [8], radio frequency-assisted, and balloon dacryocystorhinostomy. Comparisons have shown that the time required for balloon dacryocystorhinostomy was the shortest, and its success rates at 3 and 6 months after operation were the highest. In contrast, the success rates of intraoperative use of mitomycin C or 5-fluorouracil and laser-assisted dacryocystorhinostomy were found to be lower than those of standard dacryocystorhinostomy, although there were no significant differences among these methods in the incidence of complications [9].

Despite its success rates, routine endoscopic dacryocystorhinostomy has drawbacks [10]. Many factors affect the results of endoscopic dacryocystorhinostomy [11], including inappropriate location of the lacrimal sac, inadequate osteotomy, insufficient size of the ostium, inadequate marsupialization, more bleeding during the operation, cicatricial closure of the ostium, potential intranasal pathology, and abnormal anatomical structure [12]. In anatomical structure, about 2/3 of the lacrimal sac is located above the attachment point of the middle turbinate. Considering the position of the lacrimal sac and the thickness of the nasal bone, it is very important to locate the lacrimal sac correctly and remove the bone covering the lacrimal sac completely. Improper positioning of the lacrimal sac and incomplete removal of the bone will limit the exposure of the lacrimal sac and increase the chance of subsequent failure [13]. The insufficient size of the ostium is characterized by the failure to cut along the full-thickness lacrimal sac wall of the whole length, thus limiting the sac marsupialization [14]. If there is more bleeding during the operation, it will increase unnecessary time waste. And the stimulation of blood clots will also lead to the formation of granulation tissue. In addition, as already stated by others, contracture of the size of the ostium is known to occur during the healing process and ranges between 50% and 92% of the original diameter; hence, the bigger the size of the ostium is, the better the outcome should be. However, no direct correlation has been demonstrated between the neorhinostomy diameter and the final functional success rate [15]. Even so, according to our clinical experience, we think that the size of the ostium should be adequate.

In view of the factors contributing to the failure of endoscopic dacryocystorhinostomy [16], we summarized the clinical experience and improved the operation from the aspects of preoperative preparation, intraoperative bleeding control, the exposure of the lacrimal sac, incision selection, and postoperative follow-up. We perform dacryocystography before the operation to make a preliminary evaluation of the anatomical structure of the nose and the size of the lacrimal sac. This can provide some valuable information that can help predict the problems that may be encountered during the operation, adjust our psychological expectations, and deal with these problems more carefully during the operation.

We use a cotton ball that is wet with the 1 mg/ml epinephrine hydrochloride, ephedrine hydrochloride, and nitrofurazone nasal drops to fill the nasal cavity 30 minutes before the operation. The main components of furoma liquid are ephedrine hydrochloride and furacilin [17]. Ephedrine hydrochloride can directly activate vascular smooth muscle receptors and contract the blood vessels in the nasal mucosa, whereas furacilin has inhibitory effects on both Gram-positive and Gram-negative bacteria. This mixture has both anti-inflammatory and sterilization activities [18], as well as relieving congestion and edema in the nasal mucosa and reducing intraoperative bleeding. This improves surgical vision and shortens operation time, as well as provides a faster postoperative recovery.

Methylene blue is an absorbable, strongly alkaline dye [19]. After contact with healthy tissue, it enters the blood circulation, is filtered through the kidneys, and is excreted in urine. It has a relatively reduced ability to induce allergic reactions, making it a relatively safe and effective tracer. Methylene blue stains the lacrimal sac. It can not only show the boundaries between the lacrimal sac and the surrounding mucosal tissue but also make the lacrimal sac more adequately exposed and ready for marsupialization. Thus, preoperative flushing of the lacrimal duct with a diluted methylene blue solution can improve the accuracy of the operation by better determining the site of the surgical incision [20], thereby simplifying the operation and reducing surgical trauma.

A “C”-shaped incision near the lateral bone window can avoid the collapse of the anterior flap of the “I”-shaped incision and block the anastomosis of the lacrimal sac, resulting in maximal opening of the anastomosis. In addition, a gelatin sponge was applied to the junction of the lacrimal sac flap and the nasal mucosa. The expanded sponge filled the dacryocystorhinostomy, thus avoiding the curl displacement of the lacrimal sac flap and blocking the stoma. The outer edge of the nasal mucosal flap healed spontaneously with reduced adhesion to the surrounding tissue, thus effectively preventing the proliferation of granulation tissue and the formation of scar tissue in the stoma [21].

Gelatin, a product of the partial hydrolysis of natural collagen, is recognized as generally safe by the U. S. Food and Drug Administration [22]. A gelatin sponge is a type of absorbable packing material that has hemostatic and ventilatory activities as well as strong wound adsorption capacity, good histocompatibility, slow liquefaction, and absorption. Gelatin has no obvious side effects on the human body [23], is neither antigenic nor allergenic, and can meet the needs of various physiological structures within the nasal cavity. Moreover, the pressure it applies during the filling period is relatively uniform, with the degree of acceptance by patients being relatively high.

Postoperatively, the conical expansion sponge was inserted into the lacrimal sac for 1 week, effectively preventing mucosal injury and rebleeding [24]. The expansion sponge is a type of polymer expansion material that is aseptic, nontoxic, and extremely hydrophilic. It can be trimmed to a suitable size and inserted into the nasal lacrimal sac area. This sponge expands and softens rapidly after contact with water or blood, increasing local tension and applying pressure to the mucous membrane around the incision. This, in turn, reduces the proliferation of scar tissue, adhesion, and atresia of the wound and postoperative bleeding. The expanded sponge is coated with tobramycin and dexamethasone eye ointment [25], which not only has anti-inflammatory and hemostatic effects but also reduces wound edema.

The present study found that modified endoscopic dacryocystorhinostomy had a significantly higher effectiveness rate, a significantly shorter operation time, and a significantly lower intraoperative bleeding volume than routine endoscopic dacryocystorhinostomy. There were no serious complications in either group. A few patients experienced greater epistaxis during the operation and a slight headache after the operation, which improved after symptomatic support treatment.

The present study had several limitations, including its retrospective design, its selection of relatively few patients from a single institution, and its short-termfollow-up period. Randomized clinical studies with larger numbers of patients and longer follow-up times are needed to confirm these findings.

In conclusion, endoscopic dacryocystorhinostomy is currently considered the gold standard for the treatment of chronic dacryocystitis. It is reported that the success rate of this method varies. In view of the increasing correlation of this type of operation, the various types of techniques proposed rarely show their respective advantages over each other. This retrospective case study confirmed that modified seamless endoscopic dacryocystorhinostomy is one of the more successful operational types for the treatment of chronic dacryocystitis. It can significantly reduce operation time, bleeding, cicatricial closure of the ostium, and postoperative recurrence rate, while significantly improving the success rate and the postoperative lacrimal symptoms. Our report also points out that the success rate of the operation can be increased by controlling bleeding, obtaining an accurate location of the lacrimal sac, performing an adequate osteotomy, completing exposure of the lacrimal sac, and creating a sufficient size of the ostium. Last but not least, it should be emphasized that adequate preoperative preparation, meticulous endoscopic operation, and accurate postoperative follow-up are also key factors for the long-term anatomic and functional patency of the nasal ostium.

## Figures and Tables

**Table 1:** Demographic and clinical characteristics of patients who underwent modified and routine endoscopic dacryocystorhinostomy.

	Number	Age (years)	Gender (male/female)	Durationof disease (years)	OS	OD	Lacrimal sac volume (ml)
Modified group	32	51.3 ± 14.2	9/23	7.8 ± 2.9	18	14	0.62 ± 0.08
Routine group	22	48.5 ± 12.1	5/17	7.3 ± 2.5	14	9	0.59 ± 0.07
	*t*/*χ*^2^	0.755	0.198	0.658	0.117	1.423	0.755
	*P*	0.454	0.657	0.514	0.732	0.161	0.454

**Table 2:** Clinical outcomes in patients who underwent modified and routine endoscopic dacryocystorhinostomy.

	Number	Operation time (min)	Bleeding volume (ml)	6 month outcomes			
				Cure	Improvement	Ineffective	Effectiveness rate
Modified group	32	41.3 ± 12.1	12.5 ± 5.2	30 (93.75)	1 (3.12)	1 (3.12)	31 (96.88)
Routine group	22	65.4 ± 11.6	60.3 ± 8.9	13 (59.09)	2 (9.09)	7 (31.82)	15 (68.18)
*t*/*χ*^2^	—	7.312	24.883	—	—	—	6.383
*P*	—	≤0.001	≤0.001	—	—	—	0.012

## Data Availability

The data used to support the findings of this study are available from the corresponding author upon request.
